# Development of a Thermostable Freeze-Dried Live Pseudorabies Vaccine Based on the PRV Bartha-K61 Strain: Formulation Optimization, Stability Evaluation, and Preliminary Immunogenicity in Piglets

**DOI:** 10.3390/vaccines14060506

**Published:** 2026-06-04

**Authors:** Yanhong Zhao, Xiaoqing Pan, Fang Lv, Yalu Zhu, Zhen Wang, Yu Lu, Endong Bao

**Affiliations:** 1 College of Veterinary Medicine, Nanjing Agricultural University, Nanjing 210014, China; 20130990@jaas.ac.cn; 2 Institute of Veterinary Immunology & Engineering, National Research Center of Engineering and Technology for Veterinary Biologicals, Jiangsu Academy of Agricultural Sciences, Nanjing 210014, China; 3 Guotai (Taizhou) Center of Technology Innovation for Veterinary Biology, Taizhou 225300, China; 4 Livestock Research Institute, Jiangsu Academy of Agricultural Sciences, Nanjing 210014, China; pxq1611@jaas.ac.cn; 5 Nanjing Jinsiyuan Biotechnology Co., Ltd., Xuanwu District, Nanjing 210014, China

**Keywords:** pseudorabies virus, Bartha-K61, thermostable vaccine, freeze-drying, piglets, immunogenicity, vaccine formulation

## Abstract

**Background:** Pseudorabies vaccines based on the live attenuated PRV Bartha-K61 strain remain essential for controlling pseudorabies in swine, but poor thermal stability during storage and transport limits field use. **Objectives:** This study aimed to develop a thermostable freeze-dried live pseudorabies vaccine through the integrated optimization of formulation and lyophilization and to preliminarily assess its humoral immunogenicity in piglets. **Methods:** Trehalose, mannitol, and glycine were screened as candidate lyoprotectants by single-factor experiments, followed by Box–Behnken response surface optimization using viral titer retention as the response. Critical thermal parameters were determined to establish a formulation-specific lyophilization cycle. Product quality was evaluated by post-lyophilization titer, cake appearance, residual moisture, and electron microscopy, and stability was compared with a commercial freeze-dried vaccine at 2–8 °C, 25 °C, 37 °C, and 45 °C. **Conclusions:** The optimized formulation, ST005, contained 9.5% trehalose, 2.0% mannitol, and 1.5% glycine. It showed a collapse-related critical temperature of approximately −34.0 °C and a dried-product glass transition temperature of 69.1 °C, produced an intact cake with residual moisture below 3.0%, and preserved viral morphology after lyophilization. Titer loss remained below 1.0 log10 TCID_50_/mL for 24 months at 2–8 °C, 9 months at 25 °C, 21 days at 37 °C, and 9 days at 45 °C, outperforming the commercial comparator. In PRV-seronegative piglets, stored ST005 induced robust gB-specific and neutralizing antibody responses after storage under refrigerated, ambient, and accelerated conditions.

## 1. Introduction

Pseudorabies, or Aujeszky’s disease, is caused by pseudorabies virus (PRV; Suid alphaherpesvirus 1), an enveloped double-stranded DNA virus in the subfamily Alphaherpesvirinae [1,2,3]. Swine are the natural host and principal reservoir, and infection can cause fatal neurological disease in newborn piglets, respiratory disorders in growing pigs, and reproductive failure in sows, resulting in major economic losses to the pig industry [1,2,3]. PRV can also establish latent infection, which complicates eradication and long-term herd management [1,3,4]. Live attenuated vaccines based on the Bartha-K61 strain remain central to PRV prevention and control [2,3,4,5]. Bartha-K61 is a classical live attenuated PRV vaccine strain that has been widely used in prevention and control programs [2,3,4,5]. However, continued circulation of variant PRV strains has created challenges for disease control, further highlighting the importance of robust vaccine performance under field conditions [4,5]. Like many live viral vaccines, Bartha-K61-based products are highly sensitive to storage and transportation conditions, particularly temperature fluctuations that can reduce infectivity and biological activity [6,7,8].

Improving vaccine thermostability is an important objective in both human and veterinary vaccinology [6,7,8,9]. In veterinary settings, vaccines may be exposed to temperature excursions during long-distance transport, on-farm storage, and routine handling, especially where strict cold-chain management is difficult to maintain [7,8]. Therefore, the development of thermostable formulations is of considerable practical significance for improving product robustness, facilitating broader deployment, and reducing the risk of potency loss [6,7,8].

Freeze-drying, or lyophilization, is a widely used strategy to enhance the stability of biological products, including viral vaccines [10,11,12,13]. By reducing residual water content and limiting molecular mobility, lyophilization can help preserve infectivity during long-term storage [10,11,13]. However, the process itself imposes multiple stresses, including freezing-induced solute concentration, ice crystal damage, dehydration stress, and interfacial perturbation [10,12,13,14,15]. These stresses are especially relevant for enveloped viruses such as PRV, in which membrane integrity and conformational preservation of immunologically relevant proteins are essential for biological function [1,12,15].

Excipient selection is a key determinant of successful freeze-dried vaccine development [11,12,13,16]. Trehalose is widely used as a stabilizer because of its capacity to form a protective glassy matrix and preserve proteins and membranes during dehydration [17,18,19,20]. Mannitol contributes to cake structure and mechanical integrity, while glycine is often used to modulate freezing behavior and improve the physical properties of the dried matrix [21,22,23]. Because the behavior of each excipient depends on its concentration and interactions with other components in the formulation, the rational optimization of multicomponent systems is required to achieve a balance between biological protection and desirable product quality [16,21,22,23,24,25].

In parallel, lyophilization cycle design must be guided by critical thermal parameters, particularly the collapse temperature (Tc) and the glass transition temperature of the maximally freeze-concentrated system or dried product, which define the operating boundaries for robust processing [10,11,13,26]. Response surface methodology is an efficient tool for optimizing multivariable formulations because it permits factor effects and interactions to be analyzed simultaneously while minimizing the number of experiments required [27,28]. This approach is especially useful in vaccine formulation studies, where multiple quality attributes must be balanced, including infectivity retention, structural integrity, and storage stability [25,27,28].

In the present study, we developed a freeze-dried live PRV vaccine based on the Bartha-K61 strain through a stepwise strategy involving excipient screening, Box–Behnken response surface optimization, thermal characterization, lyophilization cycle optimization, and multi-temperature stability evaluation. To strengthen the translational relevance of the formulation process, preliminary immunogenicity was also assessed in PRV-seronegative piglets by measuring the responses of PRV gB-specific and neutralizing antibodies. The aim of this study was to establish a practical development framework for a thermostable live PRV vaccine suitable for further scale-up and application.

## 2. Materials and Methods

### 2.1. Virus, Antigen, and Cells

The pseudorabies virus (PRV) Bartha-K61 strain and swine testis (ST) cells used in this study were provided by the Institute of Veterinary Immunology & Engineering, Jiangsu Academy of Agricultural Sciences, Nanjing, China. The PRV antigen prepared for lyophilization had a titer of 8.0–8.5 log10 TCID_50_/mL and was confirmed to be free of bacterial contamination, mycoplasma, and adventitious viruses. ST cells were maintained in Dulbecco’s modified Eagle medium (DMEM) supplemented with 10% fetal bovine serum, 100 U/mL penicillin, and 100 μg/mL streptomycin at 37 °C in a humidified incubator with 5% CO_2_, and were used for virus propagation and titration.

### 2.2. Reagents and Instruments

Trehalose (Tre; purity ≥ 99%; lot no. T0167), D-mannitol (Man; purity ≥ 98%; lot no. M4125), and L-glycine (Gly; purity ≥ 99%; lot no. G8898) were purchased from Sigma-Aldrich (St. Louis, MO, USA) and used as candidate lyoprotectants. Dulbecco’s modified Eagle medium (DMEM), fetal bovine serum (FBS), and 0.25% trypsin-EDTA were purchased from Gibco (Grand Island, NY, USA). Penicillin–streptomycin solution (10,000 U/mL) was purchased from Solarbio (Beijing, China). A commercial PRV gB antibody ELISA kit was purchased from Keqian Biology (Wuhan, China). Karl Fischer reagents were purchased from Sinopharm Chemical Reagent Co., Ltd. (Shanghai, China). All water used in the experiments was ultrapure, prepared using a Milli-Q system and sterilized via filtration through a 0.22 μm membrane.

Residual moisture was determined using a Karl Fischer moisture titrator (V20S, Mettler-Toledo, Shanghai, China). Thermal characterization was performed using a microscopic imaging differential scanning calorimetry system (DSC3+, Mettler-Toledo, Shanghai, China) for the determination of the collapse-related critical temperature (Tc) and a differential scanning calorimeter (DSC 214 Polyma, NETZSCH, Selb, Germany) for measurement of the glass transition temperature of the dried product (Tg). Lyophilization was carried out using a laboratory freeze-dryer (SFD-1000, SIM International Co., Ltd. Seoul, Republish of Korea) Morphological characterization was performed using transmission electron microscopy (FEI Tecnai G2 F20, Hillsboro, OR, USA) and scanning electron microscopy (Zeiss Supra 40 VP, Oberkochen, Germany).

### 2.3. Virus Titration

Virus titers were determined using a micro-cytopathic effect assay in ST cells. Samples were serially diluted 10-fold and inoculated into 96-well plates containing confluent ST cell monolayers, with eight replicate wells per dilution. Plates were incubated for 5 days at 37 °C with 5% CO_2_, and cytopathic effects were recorded. Titers were calculated using the Reed–Muench method and expressed as log10 TCID_50_/mL.

### 2.4. Single-Factor Screening of Candidate Lyoprotectants

Trehalose, mannitol, and glycine were individually screened to identify suitable concentration ranges for further optimization. A basal formulation containing 5% trehalose, 2% mannitol, and 1% glycine was used as the reference formulation. During screening, the concentration of one excipient was varied while the other two were kept constant. Trehalose was tested at 2%, 5%, 8%, 10%, 12%, 14%, 16%, and 18% (*w*/*v*); mannitol at 0%, 1%, 2%, 3%, 4%, 5%, 6%, and 7% (*w*/*v*); and glycine at 0%, 0.5%, 1%, 1.5%, 2%, 2.5%, 3%, and 4% (*w*/*v*). Each condition was tested in triplicate.

Formulations were evaluated according to post-lyophilization viral titer loss, cake appearance, and reconstitution behavior. Titer retention was calculated based on infectivity values before and after lyophilization and expressed as a percentage.

### 2.5. Preparation of Protectant Formulations and Vial Filling

Protective formulations were prepared by dissolving trehalose, mannitol, and glycine in sterile water for injection. The protectant solution was mixed with PRV antigen at a 1:1 volume ratio with gentle stirring for 30 min in an ice bath (2–8 °C).

The pH was adjusted to 7.0–7.5 when necessary. Heat-stable components were sterilized with moist heat, and heat-sensitive components were sterilized by filtration through a 0.22 μm membrane. The protectant solution was mixed with PRV antigen at a 1:1 volume ratio in an ice bath with gentle stirring. The resulting mixtures were poured into 5 mL neutral borosilicate glass vials at 2.0 mL per vial, partially stoppered, and immediately transferred to the freeze-dryer. Trehalose, mannitol, and glycine were prepared as sterile excipient solutions, whereas the live PRV antigen was treated as a heat-sensitive biological component and was handled aseptically under cold conditions prior to mixing.

### 2.6. Lyophilization Procedure for Screening Experiments

For the initial excipient-screening stage, all candidate formulations were subjected to a fixed laboratory-scale lyophilization cycle so that post-lyophilization performance could be compared under the same processing conditions. The screening cycle consisted of equilibration at 4 °C for 1 h; freezing at −40 °C for 2 h; annealing at −25 °C for 3 h; refreezing at −40 °C for 2 h; and primary drying at shelf temperatures of 4 °C for 3 h, 15 °C for 5 h, and 24 °C for 4 h, followed by secondary drying at 28 °C for 6 h. Chamber pressure during drying was maintained at 10–15 Pa (Table 1).

### 2.7. Box–Behnken Response Surface Optimization

Trehalose concentration (X1), mannitol concentration (X2), and glycine concentration (X3) were selected as independent variables for optimization using a Box–Behnken design implemented in Design-Expert 12.0 software. Response surface design and model fitting were performed using Design-Expert 12.0 (Stat-Ease Inc., Minneapolis, MN, USA; https://www.statease.com/). Viral titer retention after lyophilization was used as the response variable (Y). Seventeen experimental runs, comprising twelve factorial points and five center points, were generated. Data were fitted to a quadratic polynomial model, and analysis of variance was used to assess model significance, factor effects, and lack of fit (Table 2).

### 2.8. Determination of Thermal Parameters

#### 2.8.1. Determination of Tc

The collapse-related critical temperature (Tc) of the optimized formulation was determined using a microscopic imaging differential scanning calorimetry system (DSC3+, Mettler-Toledo, Shanghai, China). Samples were subjected to controlled cooling and subsequent warming under reduced pressure, and structural changes in the frozen matrix were continuously monitored by microscopic imaging. The temperature at which visible collapse or obvious structural instability first occurred was recorded as Tc.

#### 2.8.2. Determination of Tg of the Dried Product

The glass transition temperature of the dried product (Tg) was determined with differential scanning calorimetry. Freeze-dried samples were heated to 120 °C at a rate of 5 °C/min.

### 2.9. Optimization and Validation of the Lyophilization Cycle

After selection of the practical formulation, the lyophilization cycle was further optimized specifically for ST005 based on thermal characterization and observed product performance. In contrast to the fixed screening cycle used during formulation evaluation, the final cycle was adjusted to better control the frozen matrix state, maintain product temperature within an appropriate thermal range during primary drying, and improve cake quality and batch consistency. The optimized cycle was validated in three independent batches. Product quality was assessed based on post-lyophilization viral titer, titer retention, cake appearance, reconstitution time, and residual moisture. The final optimized laboratory-scale cycle is shown in Table 6.

### 2.10. Stability Evaluation

The optimized freeze-dried PRV vaccine and a commercial freeze-dried comparator vaccine were stored at 2–8 °C, 25 °C, 37 °C, and 45 °C. Samples were collected at predefined time points, and viral titers were measured to evaluate stability. Residual moisture was also monitored during long-term storage at 2–8 °C. The commercial comparator vaccine was stored and tested under the same conditions.

### 2.11. Determination of Residual Moisture

Residual moisture content was measured with Karl Fischer titration. Samples were opened under low-humidity conditions, rapidly weighed, and analyzed; the results are expressed as percentage moisture content.

### 2.12. Transmission Electron Microscopy

Transmission electron microscopy (TEM) was used to assess the effect of freeze-drying on viral ultrastructure. Reconstituted samples were adsorbed onto carbon-coated copper grids, negatively stained with phosphotungstic acid, and examined using a transmission electron microscope (Hitachi, Tokyo, Japan) at an accelerating voltage of 120 kV. Representative micrographs were acquired at ×100,000 magnification.

### 2.13. Scanning Electron Microscopy

Freeze-dried samples were fractured to expose their internal cross-sections, mounted on specimen stubs, sputter-coated, and examined with an SEM (S3400, Hitachi, Tokyo, Japan) in secondary electron mode at 20 kV. Working distance and magnification were adjusted within the standard operating range (8.5–13.9 mm and 300×–500×, respectively) to obtain representative micrographs of the porous microstructure.

### 2.14. Preliminary Immunogenicity Evaluation in Piglets

#### 2.14.1. Animals and Grouping

Healthy 35-day-old piglets were obtained from Tianjin Ringpu Bio-Technology Co., Ltd. (Tianjin, China). Before the study, all animals underwent a 10-day acclimatization period and were housed in a clean, well-ventilated animal facility under standard husbandry conditions with free access to feed and water. Seronegativity for pseudorabies virus (PRV) was confirmed before immunization using a commercial PRV gB antibody ELISA kit (Keqian Biology, Wuhan, China) according to the manufacturer’s instructions, and only seronegative piglets were included. Piglets were then randomly assigned to six groups (n = 5 per group). Each piglet received a 1.0 mL intramuscular injection in the neck containing one vaccine dose (10^6.0^ TCID_50_), followed by a booster immunization 14 days later. The specific grouping and immunization schedule are shown in Table 3. Animals were monitored daily for general clinical condition and overt adverse reactions throughout the study. All animal procedures were conducted in accordance with the approved institutional animal care protocol.

#### 2.14.2. Serum Collection

Blood samples were collected before immunization and on Days 7, 14, 21, and 28 after primary immunization. Serum was separated with centrifugation and stored at −20 °C until analysis.

#### 2.14.3. Detection of PRV gB-Specific Antibodies

PRV gB-specific antibodies were selected as one of the primary serological markers in this study because gB is a major and highly conserved envelope glycoprotein of PRV, is strongly immunogenic, and is commonly used in commercial serological assays to evaluate vaccine-induced seroconversion. In this study, gB-specific ELISA provided a standardized and practical method for comparing retained humoral immunogenicity among vaccine preparations stored under different conditions. PRV gB-specific antibodies in piglet serum were detected using a commercial PRV gB antibody ELISA kit (Keqian Biology, Wuhan, China) according to the manufacturer’s instructions. The results were expressed as S/P values.

#### 2.14.4. Neutralizing Antibody Assay

Neutralizing antibodies were measured with a micro-neutralization assay. Serum samples were heat-inactivated at 56 °C for 30 min and serially diluted twofold. Equal volumes of diluted serum and PRV suspension containing 100 TCID_50_ were mixed and incubated at 37 °C for 1 h before addition to ST cell monolayers. Each dilution was tested in six replicate wells. After incubation for 5 days at 37 °C with 5% CO_2_, cytopathic effects were recorded. Neutralizing antibody titers were expressed as log2 values.

### 2.15. Statistical Analysis

Data are presented as mean ± standard deviation (SD). Response surface analysis and ANOVA for model fitting were performed using Design-Expert 12.0. Other statistical analyses were performed using SPSS 26.0. Response surface design and model fitting were performed using Design-Expert 12.0 (Stat-Ease Inc., Minneapolis, MN, USA; https://www.statease.com/). One-way ANOVA followed by Tukey’s multiple-comparison test was used for single-time-point comparisons, and two-way ANOVA was used for longitudinal antibody kinetics where appropriate. A value of *p* < 0.05 was considered statistically significant. Statistical analyses were performed using SPSS 26.0 (IBM Corp., Armonk, NY, USA; https://www.ibm.com/).

## 3. Results

### 3.1. Single-Factor Screening Identified Suitable Concentration Ranges for Trehalose, Mannitol and Glycine

**Figure 1:**
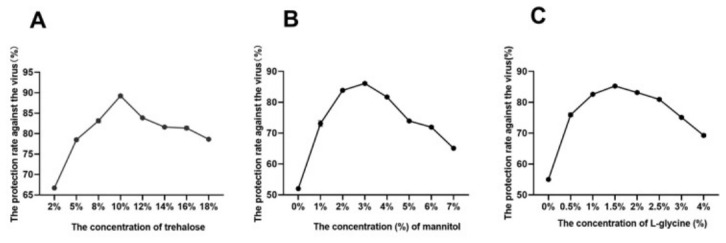
Single-factor screening of trehalose, mannitol, and glycine for freeze-dried PRV vaccine formulation. Effect of (**A**) trehalose, (**B**) mannitol and (**C**) glycine concentrations on viral titer retention after lyophilization. Data are presented as mean ± SD from three independent experiments.

Our single-factor experiments showed that trehalose, mannitol, and glycine all influenced PRV survival after freeze-drying in a concentration-dependent manner (Figure 1). Trehalose exerted a clear protective effect over the range of 2% to 10% (*w*/*v*), with viral titer retention increasing as the concentration increased, reaching a maximum of 89.23% at 10%. Further increases in trehalose concentration led to a slight decline in protection. Mannitol improved titer retention within the low-to-moderate concentration range tested, with the highest retention observed around the selected optimization range, whereas higher concentrations reduced protection. Glycine showed relatively favorable compatibility over the range of 0% to 1.5% (*w*/*v*), with the highest titer retention rate of 85.27% at 1.5%. These findings indicate that each excipient contributed to stabilization within an optimal concentration range.

### 3.2. Response Surface Optimization Predicted an Optimal Composite Formulation

**Figure 2:**
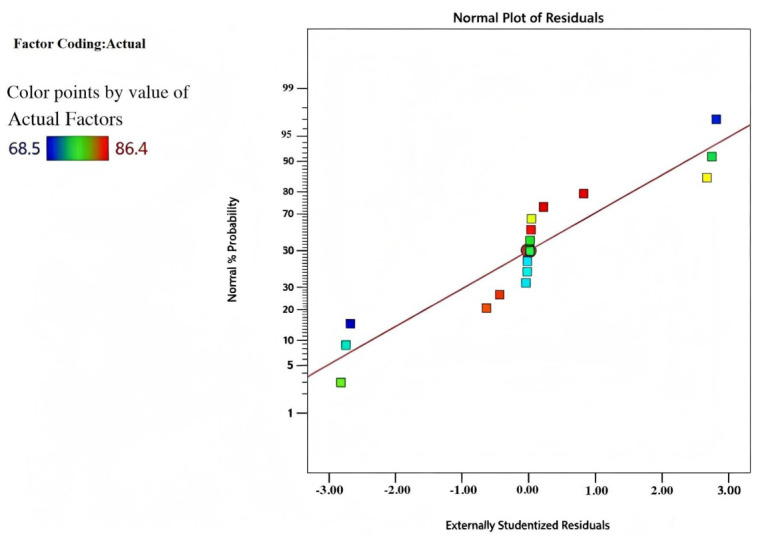
Viral titer retention rates obtained from the 17 experimental runs of the Box–Behnken design. Note: The data shown are the observed response values used for response surface model construction and formulation optimization.

The viral titer retention rates obtained from the 17 Box–Behnken experimental runs ranged from 68.5% to 86.4%, indicating that the composition of the lyophilization excipients markedly influenced the stability of the live PRV vaccine during freeze-drying (Figure 2). A quadratic polynomial model was fitted to describe the effects of trehalose (X1), mannitol (X2), and glycine (X3) on viral titer retention according to the following equation:

Y = 85.60 + 5.25X1 + 2.18X2 + 1.36X3 − 1.25X1X2 − 0.45X1X3 − 0.60X2X3 − 6.45X1^2^ − 4.20X2^2^ − 2.95X3^2^ (1)

The fitted model showed a high coefficient of determination (R^2^ = 0.9675), indicating good agreement between the observed and fitted responses (Table 4). Of the three excipients, trehalose exerted the strongest effect on viral titer retention, followed by mannitol, whereas glycine showed a comparatively smaller contribution within the tested range. In addition, the negative coefficients of the quadratic terms suggested that excessive concentrations of individual excipients could reduce the protective effect, supporting the presence of an optimal compositional range rather than a simple linear increase.

**Figure 3:**
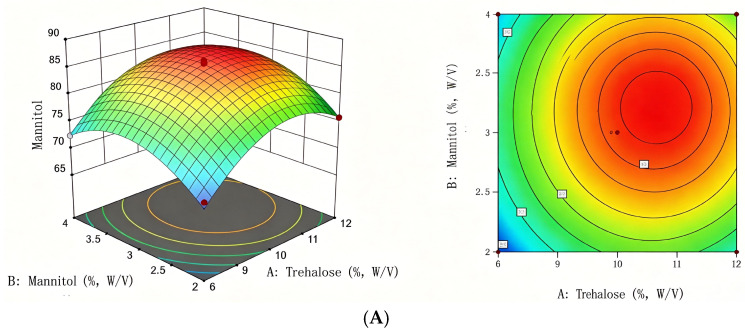

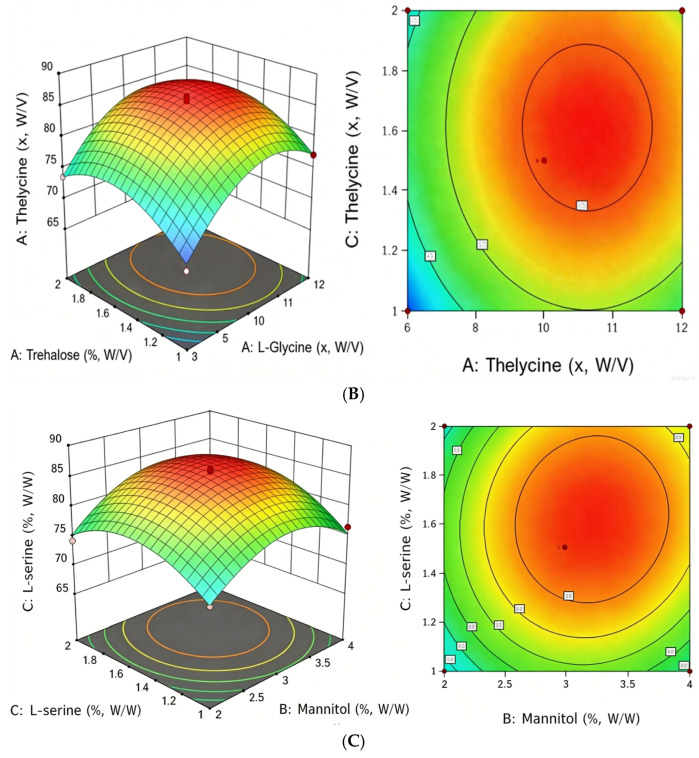
Response surface and contour plots illustrating the effects of pairwise excipient interactions on viral titer retention after lyophilization. (**A**) Trehalose and mannitol; (**B**) trehalose and glycine; (**C**) mannitol and glycine. The third variable in each panel was held at its center level.

Our response surface and contour plot analyses further illustrate the pairwise interactions between the excipients (Figure 3). The trehalose–mannitol combination showed the most evident curvature in the response surface, indicating a stronger joint effect on viral titer retention than the other factor combinations. In contrast, the interactions involving glycine were less pronounced. Overall, these analyses indicate that balanced ratios of trehalose, mannitol, and glycine are required to maximize viral stability during lyophilization.

Numerical optimization predicted that the optimal formulation consisted of 10.63% trehalose, 3.22% mannitol, and 1.62% glycine, with a predicted maximal viral titer retention rate of 86.58%. This predicted optimum was subsequently subjected to practical verification using nearby candidate formulations.

### 3.3. Practical Validation Identified ST005 as the Optimal Formulation

**Figure 4:**
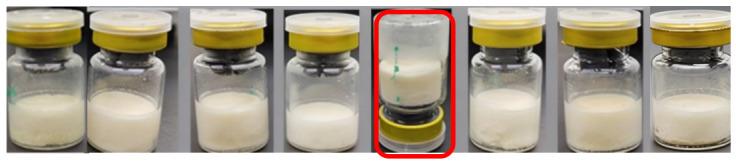
Macroscopic appearance of freeze-dried cakes prepared with different validated formulations. Note: Representative images of formulations ST001–ST008 after lyophilization are shown.

Because the mathematically predicted optimum required experimental confirmation, eight candidate formulations near the predicted optimum were prepared for practical validation by varying trehalose (9.5% or 10.0%), mannitol (0 or 2.0%), and glycine (0, 1.0%, or 1.5%) in selected combinations (detailed compositions are provided in Table S1). After lyophilization, formulations ST002, ST004, and ST005 showed relatively intact cake structures, of which ST005 exhibited the most favorable overall macroscopic appearance, characterized by a full cake, smooth surface, uniform structure, and no obvious collapse or shrinkage (Figure 4).

**Figure 5:**
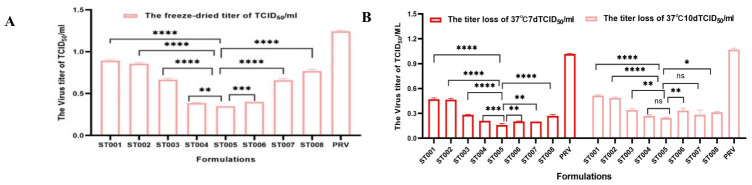
Effects of different composite protectant formulations on virus titer reduction after lyophilization and accelerated storage. (**A**) Reduction in virus titer caused by lyophilization in the eight candidate formulations. (**B**) Reduction in virus titer of the same formulations after storage at 37 °C for 7 or 10 days. Higher values indicate greater loss of infectivity (lower retained virus titer), rather than higher residual titers. Data are presented as mean ± SD. Statistical significance is indicated in the figure. ns indicates non-significant difference; * *p* < 0.05; ** *p* < 0.01; *** *p* < 0.001; **** *p* < 0.0001.

The candidate formulations were further compared in terms of post-lyophilization infectivity and short-term thermal tolerance during accelerated storage at 37 °C. In Figure 5, the plotted values represent the reduction in virus titer rather than the residual titer after storage; therefore, larger values indicate greater loss of infectivity. Accordingly, the greater titer loss observed after 10 days than after 7 days at 37 °C is consistent with progressive infectivity decay over time. Among the tested formulations, ST005 showed relatively low titer loss after lyophilization and maintained better infectivity during accelerated storage than the comparator formulations (Figure 5), indicating superior protective performance under both processing and stress conditions.

Based on the combined evaluation of cake appearance, post-lyophilization viral infectivity, and accelerated stability, ST005, containing 9.5% trehalose, 2.0% mannitol, and 1.5% glycine, was selected as the optimal practical formulation. This result was close to the response surface prediction and supported the feasibility of translating the modeled optimum into an experimentally robust formulation.

### 3.4. Thermal Analysis Guided Rational Development of the Lyophilization Cycle

Following selection of ST005 as the optimal practical formulation, thermal analysis was performed to define the process-relevant temperature boundaries for lyophilization. The collapse-related critical temperature (Tc) of ST005 was approximately −34.0 °C, whereas the glass transition temperature of the dried product (Tg) was 69.10 °C (Table 5). These parameters provide the thermal basis for rational design of the freeze-drying cycle.

In particular, the identified Tc value indicates that during primary drying, the product temperature should remain below the collapse threshold to preserve cake structure, while the relatively high Tg of the dried product supported the feasibility of subsequent drying and storage without major structural instability. On the basis of these thermal characteristics, the lyophilization program was optimized to improve drying efficiency while minimizing the risks of collapse, shrinkage, and unnecessary thermal stress.

These findings establish a formulation-specific thermal design space for ST005 and support subsequent development of an optimized laboratory-scale lyophilization cycle.

### 3.5. The Optimized Formulation and Cycle Produced a Freeze-Dried Vaccine with Favorable Quality Attributes

Application of the optimized ST005 formulation together with a formulation-specific lyophilization cycle produced freeze-dried products with favorable physical characteristics Compared with the fixed screening cycle used during preliminary excipient evaluation, the optimized program incorporated adjustments in the pre-drying steps, including shorter equilibration, deeper initial freezing, and modified refreezing conditions (Table 6), in order to better accommodate the thermal behavior of ST005 and improve the structural stability of the frozen matrix.

**Figure 6:**
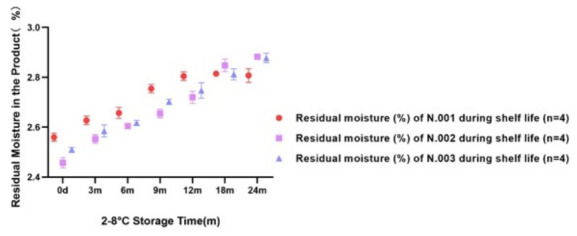
Residual moisture content of the optimized freeze-dried products during long-term storage at 2–8 °C. Residual moisture was determined using Karl Fischer titration. Data are presented as mean ± SD.

Under the optimized cycle, the resulting freeze-dried cakes were intact and well formed, with no obvious shrinkage or collapse, and the product could be readily reconstituted. The residual moisture remained below 3.0% during long-term storage at 2–8 °C (Figure 6), indicating effective dehydration and acceptable consistency of the freeze-dried product over time.

Taken together, these findings demonstrate that the combination of the optimized formulation and the formulation-specific lyophilization cycle is suitable for producing a physically stable freeze-dried PRV vaccine for subsequent stability and preliminary immunogenicity evaluation.

### 3.6. Improved Stability of the Optimized Vaccine Under Refrigerated, Ambient, and Accelerated Storage Conditions

**Figure 7:**
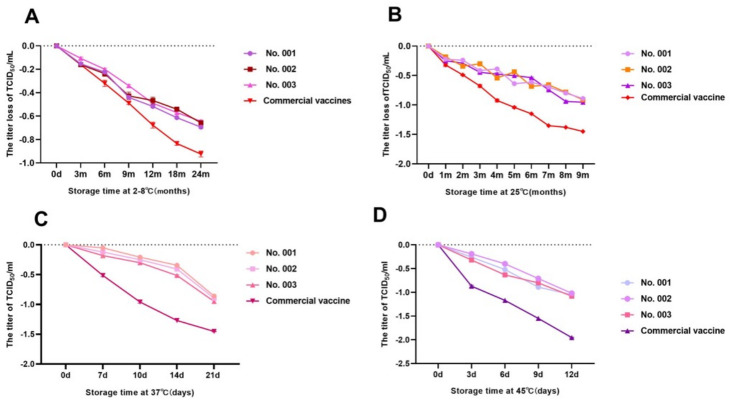
Stability profiles of the optimized freeze-dried vaccine under refrigerated, ambient, and accelerated storage conditions. (**A**) 2–8 °C for 24 months; (**B**) 25 °C for 9 months; (**C**) 37 °C for 21 days; (**D**) 45 °C for 12 days. No. 001, No. 002, and No. 003 refer to three newly prepared independent batches of the optimized freeze-dried vaccine used for confirmatory testing after formulation screening. The commercial freeze-dried vaccine was used as a comparator. These data were generated independently from the preliminary screening results shown in Figure 5.

Based on the preliminary formulation screening results shown in Figure 5, ST005 was selected as the optimized formulation for confirmatory stability evaluation. To further verify its stability performance, three newly prepared independent batches of the optimized freeze-dried vaccine (No. 001–003) were manufactured and tested under refrigerated, ambient, and accelerated storage conditions. Therefore, the data presented in Figure 7 were generated from independent confirmatory batches and are not directly comparable to the screening dataset shown in Figure 5.

Stability testing showed that the three batches of the optimized freeze-dried vaccine (No. 001–003) exhibited highly consistent titer-loss profiles under all storage conditions (Figure 7). At 2–8 °C, the decrease in infectious titer was modest over 24 months, and the loss remained below 1.0 log10 TCID50/mL throughout the study period. At 25 °C, the optimized vaccine also maintained a titer loss of <1.0 log10 TCID50/mL for 9 months, whereas the commercial vaccine declined more rapidly. Under accelerated storage, the optimized formulation remained below the 1.0 log10 TCID50/mL loss threshold for up to 21 days at 37 °C and up to 9 days at 45 °C, consistently showing better stability than the commercial vaccine. Overall, these data indicate that the optimized formulation had improved storage stability and good batch-to-batch consistency.

### 3.7. Physicochemical Analyses Supported the Protective Effect of the Optimized Formulation

**Figure 8:**
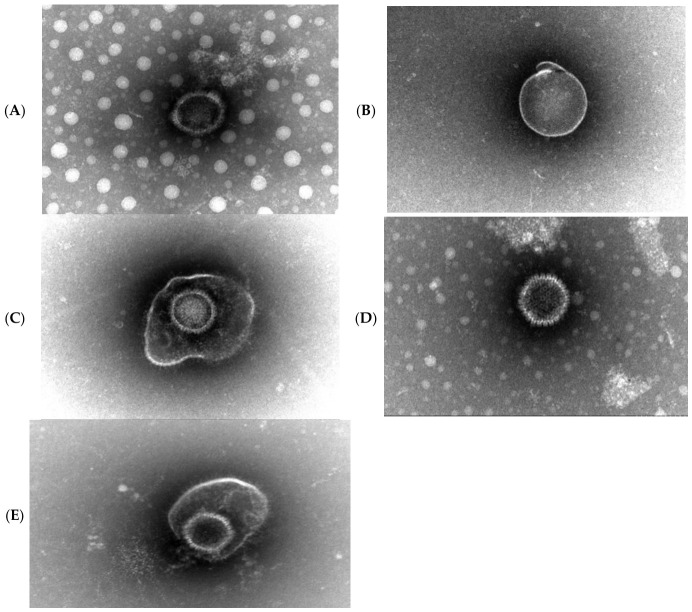
Representative TEM micrographs of reconstituted PRV particles derived from freeze-dried samples prepared with different formulations. (**A**) Without protectant; (**B**) with the complex protectant lacking trehalose; (**C**) with trehalose alone; and (**D**) with the optimized composite protectant; (**E**) PRV after lyophilization with composite protectant. Images were acquired after negative staining with phosphotungstic acid at 120 kV. Scale bar: 100 nm.

TEM analysis indicated that the optimized composite protectant preserved virion morphology more effectively after freeze-drying and reconstitution, with particles maintaining a more regular spherical shape and well-defined outlines (Figure 8D). In contrast, the unprotected sample displayed pronounced deformation and shrinkage, whereas the trehalose-only formulation and the complex protectant lacking trehalose provided only partial protection (Figure 8). Overall, these observations are consistent with the protective effect of the optimized formulation during freeze-drying.

**Figure 9:**
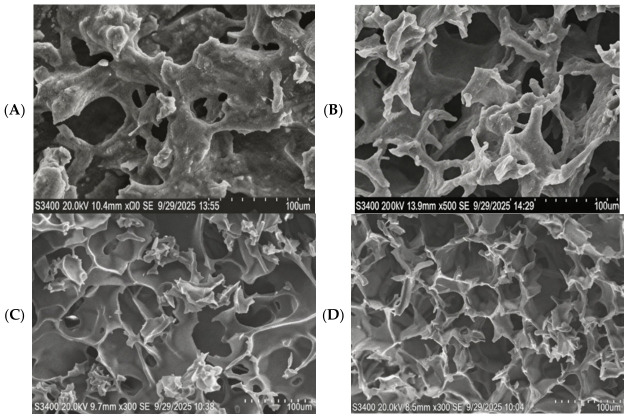
Representative SEM micrographs of freeze-dried PRV cakes prepared with different formulations. (**A**) Without protectant. (**B**) Complex protectant without trehalose. (**C**) Trehalose alone. (**D**) Optimized composite protectant. The optimized formulation exhibited a relatively uniform porous structure with no obvious collapse. Scale bar: 100 μm.

SEM analysis revealed that the optimized freeze-dried cake (Figure 9D) exhibited a porous, sponge-like morphology with a relatively uniform pore distribution and no obvious collapse. In contrast, the unprotected sample (Figure 9A) showed marked shrinkage and structural collapse, whereas the complex protectant formulation without trehalose (Figure 9B) and the trehalose-only formulation (Figure 9C) showed partial preservation of the porous network. These morphological observations were consistent with the improved stability of the optimized formulation.

### 3.8. Preserved Humoral Immunogenicity of the Optimized Freeze-Dried Vaccine in Piglets After Storage

**Figure 10:**
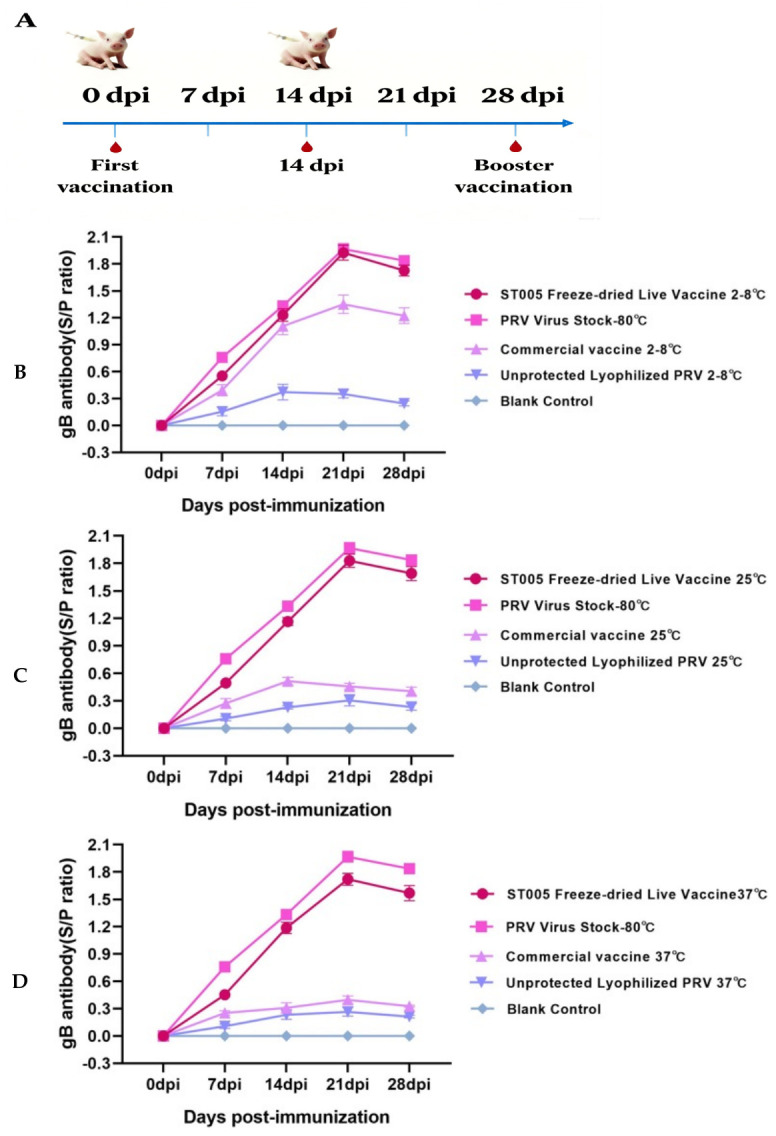
PRV gB-specific antibody responses in piglets immunized with the optimized ST005 freeze-dried vaccine after storage under different conditions. Serum gB-specific antibodies were measured by ELISA and expressed as S/P values. Data are presented as mean ± SD. (**A**) Immunization schedule. (**B**) 2–8 °C for 9 months. (**C**) 25 °C for 2 months. (**D**) 37 °C for 10 days.

To evaluate whether humoral immunogenicity was preserved after storage, piglets were immunized with the optimized ST005 vaccine stored at 2–8 °C for 9 months, 25 °C for 2 months, or 37 °C for 10 days. All piglets remained in generally good condition throughout the study, and no obvious abnormal clinical signs or vaccine-related adverse reactions were observed. No necropsy or tissue dissection was performed in this preliminary immunogenicity study. All three ST005 groups developed clear PRV gB-specific antibody responses (Figure 10). The gB-specific antibodies became detectable after primary immunization, increased markedly after booster immunization, peaked around Day 21, and remained at relatively high levels through Day 28. Among the three storage conditions, the 2–8 °C group showed the highest overall response, whereas the 25 °C and 37 °C groups showed slightly lower S/P ratios but still maintained robust antibody responses. Under all three storage conditions, the ST005 groups displayed antibody kinetics comparable to those of the fresh PRV bulk antigen group and clearly superior to those of the PBS control group. Compared with the commercial freeze-dried vaccine, the optimized ST005 groups showed comparable or higher S/P values at the major post-booster time points.

**Figure 11:**
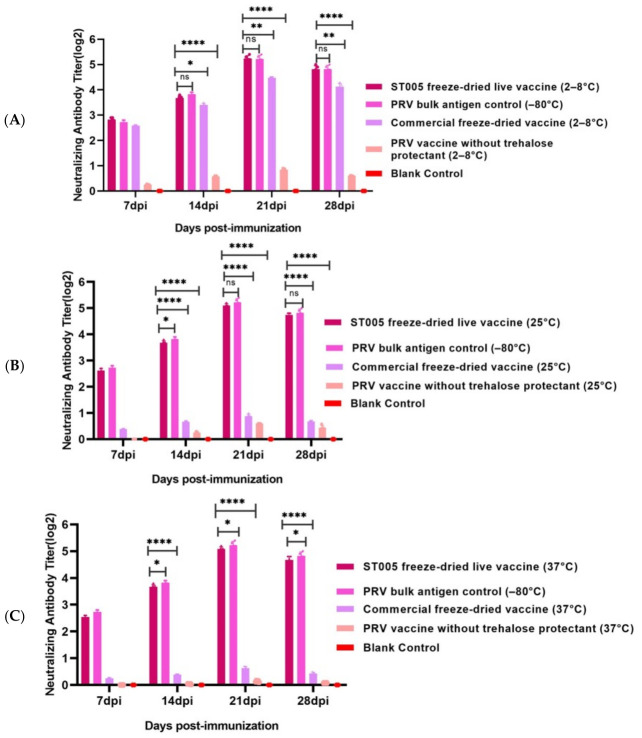
Neutralizing antibody responses in piglets immunized with the optimized ST005 freeze-dried vaccine after storage under different conditions. Neutralizing antibody titers were determined by the micro-neutralization assay and expressed as log2 values. Data are presented as mean ± SD. (**A**) 2–8 °C for 9 months. (**B**) 25 °C for 2 months. (**C**) 37 °C for 10 days. Statistical significance is indicated in the figure. ns indicates non-significant difference; * *p* < 0.05; ** *p* < 0.01; **** *p* < 0.0001.

A similar trend was observed for neutralizing antibodies (Figure 11). Neutralizing activity was detectable by Day 7 after primary immunization, increased substantially by Day 14, peaked around Day 21, and remained at relatively high levels through Day 28. In the ST005 groups, neutralizing antibody titers increased from approximately 2.6–2.8 log2 on Day 7 to approximately 5.1–5.2 log2 on Day 21 and remained around 4.7–4.8 log2 on Day 28. Although the 37 °C group showed a slight reduction in titer relative to the 2–8 °C and 25 °C groups, it still induced readily detectable neutralizing activity. These responses were close to those induced by the fresh PRV bulk antigen group and were generally higher than those induced by the commercial freeze-dried vaccine, whereas the PBS control group remained negative throughout the study.

Taken together, these findings indicate that the optimized freeze-dried vaccine retained humoral immunogenicity in piglets after prolonged refrigerated storage, medium-term ambient-temperature storage, and short-term storage under elevated-temperature conditions.

## 4. Discussion

In this study, we developed a thermostable freeze-dried live PRV vaccine through the coordinated optimization of excipient composition and lyophilization parameters, and further demonstrated the preservation of preliminary humoral immunogenicity in piglets after storage under multiple temperature conditions. The findings show that product performance depends on the combined effects of formulation design, thermal behavior, and process control rather than on any single component alone.

Trehalose exerted the strongest protective effect on post-lyophilization viral survival, which is consistent with its recognized role in preserving proteins and lipid membranes during dehydration and thermal stress [16,17,18,19,20]. Mannitol and glycine played complementary roles in improving cake structure and modulating the physicochemical properties of the frozen and dried matrix [16,21,22,23,24]. However, the effects of all three excipients were concentration-dependent, and higher concentrations were not necessarily beneficial. These results emphasize the need to balance glass-forming protection and structural support when designing freeze-dried live viral vaccines.

The response surface model demonstrated that trehalose, mannitol, and glycine all significantly affected viral titer retention, with the interaction between trehalose and mannitol being particularly notable. Although the mathematically predicted optimum differed slightly from the final practical formulation, our experimental validation demonstrated that ST005 achieved the most favorable balance between infectivity retention, cake appearance, and thermal tolerance. This finding reinforces the importance of combining statistical optimization with direct product evaluation in vaccine formulation studies.

Thermal analysis was critical for lyophilization cycle design. The measured critical thermal limit, together with the overall thermal characteristics of the formulation, provided clear guidance for freezing and primary and secondary drying [6,8]. By maintaining product temperature within an appropriate range during processing, we obtained freeze-dried cakes with favorable appearance, low residual moisture, and reproducible infectivity retention. These results support the utility of thermally guided process development in improving the robustness of freeze-dried vaccine products [13,26,29,30].

The optimized formulation exhibited improved stability under refrigerated, ambient, and accelerated temperature conditions. The retention of acceptable infectivity for 24 months at 2–8 °C and 9 months at 25 °C suggests meaningful gains in storage flexibility, while the improved tolerance to temperatures of 37 °C and 45 °C indicates better resilience to temporary temperature excursions. Such robustness is particularly relevant for veterinary vaccines, which may be exposed to variable transport and field-handling conditions [7,8,9,10].

Residual moisture and morphological analyses further support the stability data. Residual moisture remained below 3.0%, which is generally compatible with maintenance of a stable glassy matrix [25]. TEM showed that the optimized formulation better preserved viral ultrastructure during freeze-drying, and SEM showed the formation of a porous and structurally coherent cake. These characteristics were consistent with the improved reconstitution behavior and storage stability of the final product.

Importantly, the present study also demonstrated that improved physicochemical stability was accompanied by the preservation of preliminary biological function in the target species. The optimized ST005 formulation retained the ability to induce robust PRV gB-specific and neutralizing antibody responses in piglets after storage at 2–8 °C for 9 months, 25 °C for 2 months, and 37 °C for 10 days. PRV gB-specific antibodies were used as a practical and standardized serological marker because gB is a major conserved envelope glycoprotein of PRV and a common target in commercial antibody assays. In addition, gB-specific antibody detection was evaluated together with neutralizing antibody titers, thereby providing both antigen-specific and functional measures of retained humoral immunogenicity after storage. These responses were close to those induced by the fresh PRV bulk antigen and were generally stronger than those induced by the commercial freeze-dried comparator. Notably, although the 37 °C group showed a slight reduction in antibody levels relative to the 2–8 °C and 25 °C groups, it still elicited clear and readily detectable humoral responses. This observation suggests that the formulation and process preserved both viral infectivity and antigenic integrity relevant to humoral immune induction, even after short-term storage under elevated-temperature conditions.

This study has several limitations. First, all work was performed at the laboratory scale, and additional studies will be required to assess process robustness during pilot-scale and manufacturing-scale transfer. Second, although preliminary humoral responses were assessed in piglets, full protective efficacy and post-storage challenge studies were beyond the scope of this work. Third, cellular immune responses were not evaluated and should be included in future studies to provide a more comprehensive assessment of vaccine-induced immunity. Fourth, the detailed molecular mechanisms underlying the synergistic stabilization effects of the selected excipients remain to be investigated. Nevertheless, the present study provides a coherent framework for thermostable live vaccine development by linking formulation optimization, thermal characterization, stability evaluation, and preliminary target-animal validation.

Overall, our data indicate that rational excipient selection combined with thermally informed lyophilization can substantially improve the stability of a live PRV vaccine without impairing preliminary humoral immunogenicity in piglets. These findings support the further development of thermostable live veterinary vaccines with improved logistical flexibility.

## 5. Conclusions

A thermostable freeze-dried live pseudorabies vaccine based on the PRV Bartha-K61 strain was successfully developed through the integrated optimization of formulation composition and lyophilization parameters. The selected formulation, ST005, exhibited a favorable cake appearance, low residual moisture, improved preservation of viral morphology, and enhanced stability under refrigerated-, ambient-, and elevated-temperature conditions. Preliminary immunogenicity evaluation in piglets showed that the optimized freeze-dried vaccine retained the ability to induce robust PRV gB-specific and neutralizing antibody responses after storage at 2–8 °C for 9 months, 25 °C for 2 months, and 37 °C for 10 days. These results provide a practical basis for the further development of thermostable live PRV vaccines.

## Figures and Tables

**Table 1:** Fixed screening lyophilization cycle used for single-factor excipient evaluation.

Process Step	Shelf Temperature (°C)	Chamber Pressure (Pa)	Holding Time	Purpose
Equilibration	4	-	1 h	Temperature equilibration before freezing
Freezing	−40	-	2 h	Complete freezing of the product
Annealing	−25	-	3 h	Crystal growth and matrix stabilization
Refreezing	−40	-	2 h	Re-establishment of frozen structure
Primary drying, stage 1	4	10–15	3 h	Initial sublimation
Primary drying, stage 2	15	10–15	5 h	Continued sublimation
Primary drying, stage 3	24	10–15	4 h	Completion of primary drying
Secondary drying	28	10–15	6 h	Removal of bound water

**Table 2:** Box–Behnken experimental design matrix.

Run	X_1_: Trehalose (%)	X_2_: Mannitol (%)	X_3_: Glycine (%)
1	−1 (8)	−1 (2)	0 (1.5)
2	+1 (12)	−1 (2)	0 (1.5)
3	−1 (8)	+1 (4)	0 (1.5)
4	+1 (12)	+1 (4)	0 (1.5)
5	−1 (8)	0 (3)	−1 (1)
6	+1 (12)	0 (3)	−1 (1)
7	−1 (8)	0 (3)	+1 (2)
8	+1 (12)	0 (3)	+1 (2)
9	0 (10)	−1 (2)	−1 (1)
10	0 (10)	+1 (4)	−1 (1)
11	0 (10)	−1 (2)	+1 (2)
12	0 (10)	+1 (4)	+1 (2)
13 (center point)	0 (10)	0 (3)	0 (1.5)
14 (center point)	0 (10)	0 (3)	0 (1.5)
15 (center point)	0 (10)	0 (3)	0 (1.5)
16 (center point)	0 (10)	0 (3)	0 (1.5)
17 (center point)	0 (10)	0 (3)	0 (1.5)

Abbreviations: X_1_, trehalose concentration; X_2_, mannitol concentration; X_3_, glycine concentration. −1 = low level, 0 = center level, 1 = high level.

**Table 3:** Piglet grouping and immunization schedule for preliminary immunogenicity evaluation.

Group	Sample/Immunogen	Storage Condition	Number of Piglets	Volume
1	ST005 freeze-dried vaccine	2–8 °C for 9 months	5	10^6.0^ TCID_50_/dose
2	ST005 freeze-dried vaccine	25 °C for 2 months	5	10^6.0^ TCID_50_/dose
3	ST005 freeze-dried vaccine	37 °C for 10 days	5	10^6.0^ TCID_50_/dose
4	Fresh PRV bulk antigen	Freshly prepared	5	10^6.0^ TCID_50_/dose
5	Commercial freeze-dried vaccine	According to manufacturer’s instructions	5	1 dose
6	PBS control	/	5	/

Note: Piglets were immunized intramuscularly in the neck with 1.0 mL per dose. A booster immunization was administered 14 days after the primary vaccination.

**Table 4:** Summary of the fitted quadratic polynomial model for viral titer retention.

Item	Result
Experimental design	Three-factor, three-level Box–Behnken design
Number of experimental runs	17
Response variable	Viral titer retention rate (%)
Model type	Quadratic polynomial model
Regression equation	Y = 85.60 + 5.25X1 + 2.18X2 + 1.36X3 − 1.25X1X2 − 0.45X1X3 − 0.60X2X3 − 6.45X1^2^ − 4.20X2^2^ − 2.95X3^2^
Coefficient of determination (R^2^)	0.9675
Main influential factor	Trehalose (X1)
Secondary influential factor	Mannitol (X2)
Relative contribution of glycine	Lower than trehalose and mannitol within the tested range
Predicted optimal formulation	10.63% trehalose, 3.22% mannitol, and 1.62% glycine
Predicted maximal viral titer retention	86.58%

Abbreviations: X1, trehalose concentration; X2, mannitol concentration; X3, glycine concentration.

**Table 5:** Critical thermal parameters of the optimized ST005 formulation.

Parameter	Value (°C)	Method	Interpretation
Tc	−34.0	Freeze-drying microscopy	Collapse-related critical temperature
Tg	69.10	Differential scanning calorimetry	Glass transition temperature of the dried product

Abbreviations: Tc, collapse-related critical temperature; Tg, glass transition temperature of the dried product.

**Table 6:** Optimized laboratory-scale lyophilization cycle for the ST005 freeze-dried PRV vaccine.

Process Step	Shelf Temperature (°C)	Chamber Pressure (Pa)	Holding Time
Equilibration	4		0.5 h
Freezing	−45		2 h
Annealing	−25		2 h
Refreezing	−38		4 h
Primary drying, stage 1	4	10–15	3 h
Primary drying, stage 2	15	10–15	5 h
Primary drying, stage 3	24	10–15	4 h
Secondary drying	28	10–15	6 h

Note: This table shows the final laboratory-scale lyophilization cycle optimized for the ST005 formulation after thermal characterization. Unlike the fixed screening cycle used for excipient evaluation (Table 1), this program was formulation-specific and was used for preparation of the final freeze-dried PRV vaccine.

## Data Availability

The data supporting the findings of this study are available from the corresponding author upon reasonable request, subject to institutional data availability policies.

## Supplementary Materials

The following supporting information can be downloaded at: https://www.mdpi.com/article/10.3390/vaccines14060506/s1, Table S1: Candidate composite formulations designed for practical validation based on response surface optimization.

## Abbreviations

PRV, pseudorabies virus; TEM, transmission electron microscopy; SEM, scanning electron microscopy; Tc, collapse-related critical temperature; Tg, glass transition temperature of the dried product; TCID_50_, 50% tissue culture infectious dose; ANOVA, analysis of variance; PBS, phosphate-buffered saline; ELISA, enzyme-linked immunosorbent assay; SD, standard deviation.
