# Supplementary material for: Development of a Thermostable Freeze-Dried Live Pseudorabies Vaccine Based on the PRV Bartha-K61 Strain: Formulation Optimization, Stability Evaluation, and Preliminary Immunogenicity in Piglets
Source: Vaccines (Basel). 2026 Jun 4;14(6):506. doi: 10.3390/vaccines14060506 (PMC13308344; doi:10.3390/vaccines14060506)
Supplement: Supplementary file 1 [file vaccines-14-00506-s001.zip › vaccines-4291320-supplementary.pdf]

Table S1. Candidate composite formulations designed for practical validation based on response surface optimization.

Formulation	Trehalose (%)	Mannitol (%	L-Glycine (%)
ST001	9.5	2	1.0
ST002	10	2	1.0
ST003	9.5	2	/
ST004	10	2	/
ST005	9.5	2	1.5
ST006	10	2	1.5
ST007	9.5	/	1.5
ST008	10	/	1.5

Note: These formulations were selected for practical verification based on the response surface optimization results.
